# An Exploratory Assessment of Applying Risk Management Practices to Engineered Nanomaterials

**DOI:** 10.3390/ijerph16183290

**Published:** 2019-09-07

**Authors:** Ivo Iavicoli, Veruscka Leso, Marco Piacci, Dante Luigi Cioffi, Irina Guseva Canu, Paul A. Schulte

**Affiliations:** 1 Department of Public Health, Section of Occupational Medicine, University of Naples Federico II, Via S. Pansini 5, 80131 Naples, Italy (V.L.) (M.P.) (D.L.C.); 2 Institute for Health and Work (IST), Biopole, Route de la Corniche 2, CH-1066 Epalinges-Lausanne, Switzerland; 3 National Institute for Occupational Safety and Health, Centers for Disease Control and Prevention, 1150 Tusculum Avenue, MS C-14, Cincinnati, OH 45226, USA

**Keywords:** nanomaterials, industry, risk assessment, risk management, preventive measures, survey

## Abstract

The widespread industrial application of nanotechnology has increased the number of workers exposed to engineered nanomaterials (ENMs), but it is not clear to what extent prevention guidance is practiced. Our aim was to explore the extent that companies manufacturing and/or using ENMs apply risk assessment and management measures. Thirty-four companies were surveyed with an international 35-item questionnaire investigating company and workforce features, types of ENM handled, and risk evaluation and preventive measures adopted. Among participating companies, 62% had a maximum of 10 employees. Metal-based nanomaterials were most frequently identified (73%). Environmental monitoring was performed by 41% of the companies, while engineering exposure controls were approximately reported by 50%. Information and training programs were indicated by 85% of the sample, only 9% performed specific health surveillance for ENM workers. Personal protective equipment primarily included gloves (100%) and eye/face protection (94%). This small-scale assessment can contribute to the limited amount of published literature on the topic. Future investigations should include a greater number of companies to better represent ENM workplaces and a direct access to industrial settings to collect information on site. Finally, deeper attention should be paid to define standardized frameworks for ENM risk assessment that may guide nano-specific preventive actions.

## 1. Introduction

Nanotechnology involves the production of 1–100-nm-sized materials exploited in various production and industrial fields, such as structural engineering, electronics, consumer products, energy production and storage, environmental quality, and biomedicine sectors [1]. This widespread application relies on the unique technological properties of engineered nanomaterials (ENMs), including mechanical hardness, scratch, and corrosion resistance; thermal insulation and heat resistance; and optical, electronic and magnetic properties [2]. However, the specific physico-chemical properties of ENMs that make them so attractive for commercial applications may in turn influence their biological reactivity and raise concerns of possible ENM health effects [3,4,5,6]. From an occupational health perspective, this is an important issue, considering the widespread application of ENMs, the increasing number of workers expected to become exposed to such materials throughout the products life-cycles, only preliminary data being available concerning any possible early effects of ENMs on involved workers, as well as the still-limited knowledge concerning the toxicokinetic and dynamic behavior of such xenobiotics in animal models [5]. Consequently, in the early 2000s, international authoritative agencies issued guidance to take precautions when using ENMs [2,7,8,9,10].

However, although some regulations appear necessary to guide ENM health and safety procedures, there are some differences of views among stakeholders involved in the different nanotechnology areas (i.e., academics, industry, employees, non-governmental organizations and regulators) on whether policies should be evidence-based, precautionary- or business-oriented, or voluntary or top-down controlled [11,12]. This has led to ENM governance generally falling under regulations with respect to the specific ENM field of application, i.e., food or cosmetic products, or under voluntary guidance from governmental agencies or trade organizations [12,13], thus making the industry directly responsible for safe ENM work-practices [11]. 

Therefore, it is important to address the type and extent of risk assessment and management approaches initiated by industries. Challenges in determining this information come from limitations in gaining access to ENM companies and their workforce to conduct on-site evaluations, which prevent acquiring adequate information concerning the organizational aspects of ENM companies, the global amount of the ENM workforce, as well as awareness on possible risks from ENM exposure and measures adopted for the protection of workers. To overcome such knowledge-gaps, a survey-research questionnaire aimed at collecting self-reported information on ENM industrial practices, risk assessment and precautionary management strategies, was developed. To date, few self-reported surveys have been performed in Europe [11,12,14] and the US [15] in companies operating in research, development, production, and use of ENMs. Those studies’ primary goal was to verify the industrial adherence to the currently-available precautionary risk-management guidance, and the effectiveness and critical issues of such practices in promoting the overall protection of the workforce [12]. However, differences existing in the multiple ENM working environments make the standardization and broad coverage of the results a challenging issue.

In this context, to further identify how companies that manufacture or use ENMs apply risk assessment and management measures, a pilot questionnaire evaluation was conducted. The topics in the questionnaire included general information regarding the characteristics of the companies, how the ENM workforce was dispersed in the industry, types of ENM handled, risk evaluation, and preventive measures adopted. The purpose of this assessment was to reveal information about relevant practices and perceived weaknesses in currently-employed preventive strategies with the aim to point out some interesting data as well as criticisms that may need future investigation to be able to achieve more solid scientific evidence for guiding the adoption and implementation of specifically-focused ENM governance. 

## 2. Materials and Methods 

The international online survey was based on a questionnaire developed by the Scientific Committee on “Nanomaterial Workers’ Health” of the International Commission on Occupational Health (ICOH). The questionnaire utilized input based on a publicly-available draft questionnaire from the US National Institute for Occupational Safety and Health (NIOSH) [16].

The survey consisted of 35 questions in the English language, focused on different thematic areas. Among those, the general company features were explored, including nationalities of the respondent operating unit, commercial sector in which ENMs or nano-enabled products were involved, and total amount of employed workers and number of employees assigned to nanomaterial-related tasks. Specific questions addressed the ENM chemical composition, the physical status in which ENMs were handled, and the total amount used during a typical work-day. This information was deemed necessary to assess ENM hazards and possible routes and entities of exposure. Risk management procedures including resources used to acquire information about handling or safety and health practices regarding ENMs were addressed in specific survey questions. Finally, companies were also asked to indicate which, among a wide variety of engineering controls, good practices at work, as well as personal protective equipment, have been adopted as primary preventive measures to reduce workers’ exposure. Health and safety, workplace risk-training programs, as well as health surveillance plans, as key elements in ENM workers’ protection, were also explored. 

To identify possible companies to be included in our study, we searched for representative web-databases interested in industrial ENM innovation or in the nanotechnology sector. Seven website databases were employed to collect information and email-contact of ENM-involved industries. Companies of all nationalities reported in the databases were all considered suitable for inclusion if they were involved in working with ENMs. Companies operating in the US were excluded as they may be included in a similar effort being proposed by NIOSH [17]. No control group was considered due to the exploratory nature of the study. This preliminary search identified a total of 2029 companies. The survey was open from 7 July 2017 to 2 July 2018. The first contact resulted in 289 email delivery failures, and the corresponding companies were excluded from the survey sample, which left 1740 facilities remaining. During the survey year, these companies were solicited to answer the questionnaire once or twice a month for a total of 15 contacts via e-mail or through their website contact forms. The link to access the questionnaire was sent accompanied by an informative letter including a simple, although detailed explanation of the aim of the study, and some brief details concerning the time required to complete the compilation (about 30 min) and the need for a person with a great competence/knowledge of the company job processes and the health and safety policies to answer the questionnaire. The participating companies had to provide their informed consent before completing the questionnaire. The answers provided through the internet form were automatically exported into an Excel file to avoid the misreporting of data. To ensure data confidentiality, all collected information was anonymized before any analysis, by replacing company names with a code and protecting all electronic records from unauthorized access, as only two of the authors had access to the export dashboard and knew the password set to protect the Excel file. A quantitative analysis was performed on the reported answers, and for each question, the absolute number of responsive companies and percentage of the total sample were calculated. Results have been analyzed and reported in an anonymous format so that no external person can identify any company who took part in the survey.

## 3. Results

A total of 45 companies provided a response to our mail-contact. This represents a 2.58% response rate. Out of these companies, 34 responded completing the questionnaire. Eleven companies declared to not be involved in producing and/or processing ENMs, providing no answer to the questionnaire, and were not included in the analyses. The following paragraphs summarize the results and include a descriptive analysis of the main thematic areas addressed in the survey.

### 3.1. Company Characteristics

**Figure 1:**
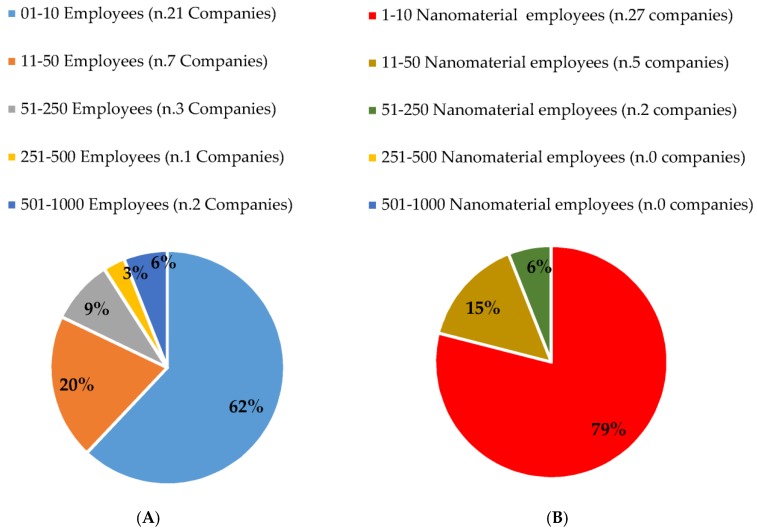
Workers employed in respondent companies. (**A**) Percentage of respondent companies based on the number of employed workers; (**B**) Percentage of respondent companies based on the number of nanomaterial involved workers.

**Figure 2:**
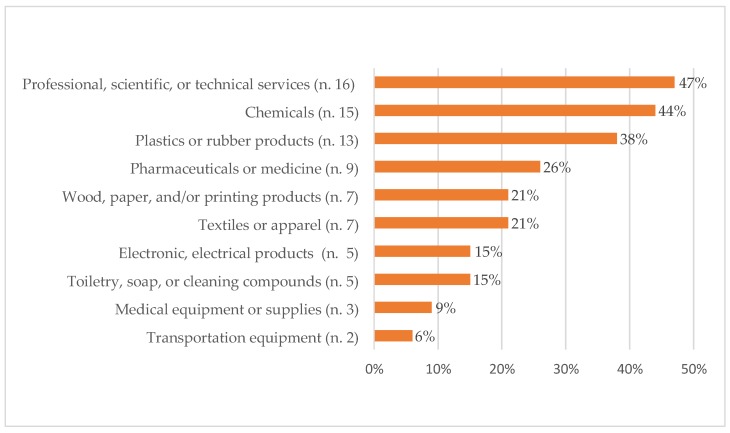
Commercial sectors where engineered nanomaterials or nano-enabled products/services were intended to be used (*n* = number of respondent companies); percentages.

Among respondent companies, 26 were from Europe, 5 from Asia and 1 from North America (Table 1). Most of the respondent companies had a maximum of 10 workers (62%) (Figure 1A). Concerning the number of ENM workers, 79% (*n* = 27) of the respondent companies indicated 1–10 employees as directly involved with handling ENMs (Figure 1B). Commercial sectors where the ENMs or nano-enabled products/services were intended to be used, primarily included the professional, technical or scientific services (*n* = 16; 47%); chemical fields (*n* = 15; 44%); and plastics and rubber product sectors (*n* = 13; 38%) (Figure 2). Specific activities related to ENMs included development (*n* = 30; 88%), manufacturing (*n* = 18; 53%), and laboratory scale-up (*n* = 12; 35%). Concerning ENM application activities, 56% of the respondent companies (*n* = 19) were dedicated to the development of nano-enabled applications or products and 35% to ENM incorporation into other products (*n* = 12). A smaller percentage of respondent companies reported being involved in packaging and distributing ENMs (*n* = 6; 18%), providing services using nano-enabled tools (*n* = 6; 18%), as well as developing instruments for the manufacture, characterization or detection of ENMs (*n* = 3; 9%). Physico-chemical characterization of ENMs was performed by 16 of the respondent companies (47%).

### 3.2. Nanomaterial Features

Concerning quantitative aspects of ENM production and application, metal or metal oxide-based ENMs were among the most frequently employed materials with silver and gold nanoparticles (NPs) employed in 44% (*n* = 15) and 41% (*n* = 14) of all the respondent companies, respectively, followed by titanium dioxide (*n* = 10; 29%) and zinc oxide NPs (*n* = 7; 21%). Among the carbon-based ENMs, graphene was the most-used substance (*n* = 9; 26%), whereas single and multi-walled carbon nanotubes were used by 15% (*n* = 6) and 6% (*n* = 2), respectively. Regarding the physical state in which ENMs were used, they were generally employed in the form of a liquid suspension (*n* = 25; 73%); freely movable solids (*n* = 17, 50%); suspended in a matrix (*n* = 16, 47%); or as solids embedded, bound, or fixed in a material or product (*n* = 13; 38%). Only 3% of respondent companies worked ENMs in the form of aerosols. Concerning the approximate quantity of ENMs handled, 27 companies reported using less than a kg per day (79%), while 7 reported to handle more than 1 kg of ENMs per day (21%).

### 3.3. Development of Health and Safety Practices

Respondent companies reported using a great number of resources to acquire information about handling ENMs, as well as guidance on nano-specific health and safety practices. These included industry, scientific, and professional meetings, conferences, or tradeshows (*n* = 23; 68%); governmental publications and materials (*n* = 22; 65%); product manufacturer information (*n* = 20; 59%); scientific, professional or industry publications (*n* = 20; 59%); websites, blogs, and internet search engines (*n* = 19; 56%); and informal discussions with professional contacts or peers (*n* = 18; 53%). When specific safety and health practices or guidelines for ENMs were not available, other general chemicals in the workplace were reported to have been considered as surrogate substances to inform ENM risk-management strategies from the majority of the respondent companies (*n* = 25; 73%). Acquired knowledge was reported to update health and safety practices in the workplace (82%), and company policies for ENM handling (71%), as well as the production of informative material ad hoc (Table 2). Forty-four percent of the companies indicated that some changes in the work processes were also introduced according to the information collected, and this was also incorporated both into training materials (41%), as well as in product information materials, like safety data sheets (44%). In some cases, the acquired knowledge had stimulated changes in products (26%) as well as in workplace processes (44%) (Table 2). Eighty-five percent of companies reported producing documents related to ENMs, including scientific publications (62%), journal articles (45%), as well as articles for trade magazines (28%). Comments on public policies; government documents; industry or materials standards; and professional, scientific or trade associations were provided by approximately 14% of the companies, respectively. Instructions for product use or guidance for ENM measurements, information on company’s standard operating procedures or practices, as well as customer information among company’s products or services were provided by 34%, 28% and 55% respectively of the survey sample.

### 3.4. Instituted Health and Safety Programs

Up to 70% of the respondent companies indicated that they had instituted a series of general chemical hygiene practices as part of the health and safety programs. However, when the specific approaches for ENMs were assessed, nano-focused preventive measures were less frequently reported. These primarily pertained to: the identification of processes or job tasks where workers could be exposed to ENMs (*n* = 18; 53%), determination of routes of exposure (*n* = 16; 47%), the evaluation of new processes and procedures for hazards identification (*n* = 17; 50%), the use of exposure control measures (*n* = 19; 56%), as well as spill cleanup procedures (*n* = 16; 47%) and waste management and disposal procedures (*n* = 17; 47%). Other practices were indicated to be used in a smaller percentage of the respondent companies (Table 3).

### 3.5. Exposure Monitoring

Monitoring the workplace for ENM levels represents a distinguishing feature of suitable health and safety programs in nano-industry. When ENM exposure assessment was explored, the majority of the respondent companies (*n* = 20; 59%) declared that environmental monitoring was not performed. Among the remaining 41% of the respondent companies (*n* = 14), different methodological techniques were reported to be used to assess exposure levels. These included direct reading particle counters (*n* = 7; 50%), filter-based air sampling for electron microscopy (*n* = 5; 36%), filter-based air sampling for mass (*n* = 3; 21%), and wipe sampling (*n* = 2; 14%). Among the 14 companies performing exposure measurements, 7 (50%) declared a regular schedule for environmental monitoring (e.g., annually). In the other cases, occasions for exposure assessment included initial process start-up (*n* = 4; 29%), changes in processes or controls (*n* = 6, 43%), as well as upset conditions, such as a response to a spill or similar unanticipated accidental events (*n* = 2; 14%).

### 3.6. Exposure Control 

**Figure 3:**
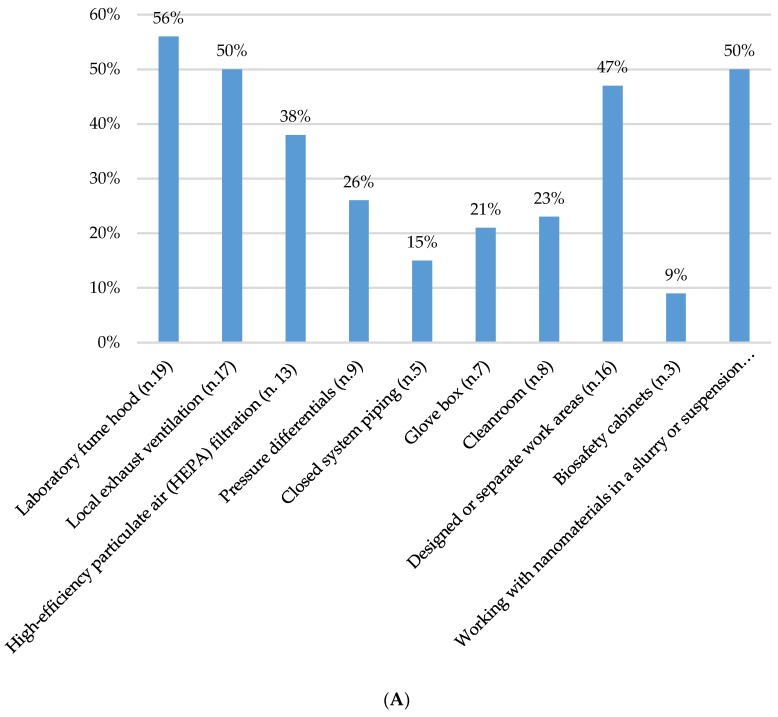

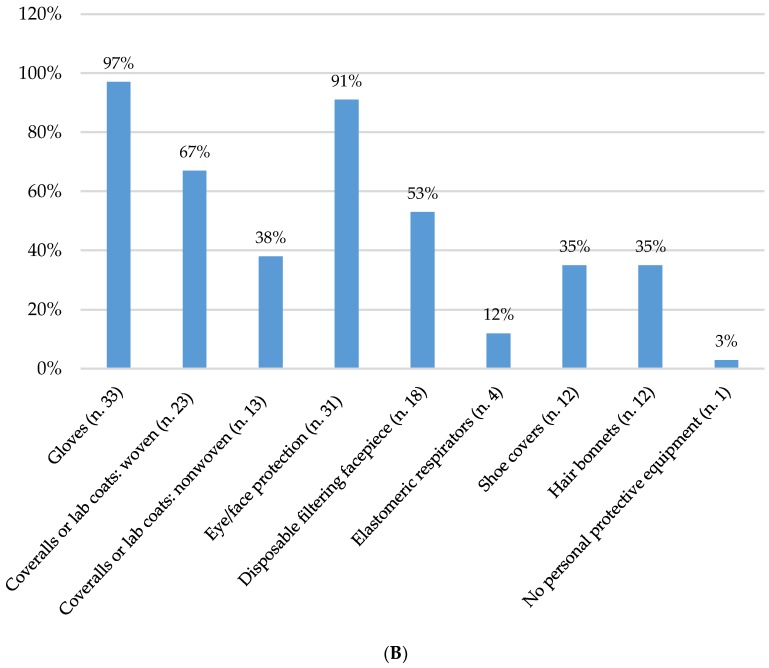
Engineering controls and personal protective equipment used for exposure control. (**A**) Collective protective devices (*n* = number of respondent companies); percentages; (**B**) Personal protective equipment (*n* = number of respondent companies); percentages.

Multiple engineering control strategies have been employed to control or reduce workers’ exposure to ENMs. Laboratory fume hoods characterized the primary air contaminant engineering control adopted, as 56% (*n* = 19) of the respondent companies reported their use. Additionally, a number of respondent companies reported using local exhaust ventilation, (*n* = 17; 50%) and HEPA filtration for air exhausted from a process room to outside a building (*n* = 13; 38%) to remove airborne ENMs. Many respondent companies (*n* = 16; 47%) also indicated using separate work areas (e.g., control room) for handling ENMs or handling ENMs in a slurry or in suspension to reduce possible environmental dispersion (*n* = 17; 50%). Cleanrooms utilized in a controlled environment to maintain a low particle content were adopted in 23% of cases (*n* = 8). As for enclosed-systems, 21% of the respondent companies (*n* = 7) reported using glove boxes for a controlled handling of substances to avoid worker exposure (Figure 3A).

### 3.7. Administrative Controls

Health and safety programs include the use of administrative controls, which may include training and health surveillance plans. A total of 85% of respondent companies reported providing information and training to employees with regular or occasional contact with ENMs. Training programs included the type of ENMs and general ENM awareness and routes of exposure; where to find information about the safety and health practices; use or maintenance of engineering controls; procedures for spill clean-up, waste management, and disposal procedures; and methods for reporting hazards, illnesses, and injuries. Training was provided by several means including: informal training by colleagues (*n* = 17; 59%), internal formal training (*n* = 12; 41%), and external training or consultants (*n* = 8; 28%). Workers awareness and knowledge concerning ENM was assessed in 62% (*n* = 21) of the respondent companies through random inspections or periodic surveys, the 62% (*n* = 13) and 48% (*n* = 10) of this subgroup, respectively. Among the 12 companies (35% of the survey respondent sample) that performed health surveillance activities, only three companies developed specific protocols for possible ENM-related health risks.

### 3.8. Personal Protective Equipment (PPE)

Regarding PPE adopted by ENM workers, all but one company reported the use of some type of PPE. This included gloves (*n* = 33, 97% of the total sample), woven lab coats (*n* = 23; 67%), nonwoven lab coats or coveralls (*n* = 13; 38%), eye and face protection (*n* = 31; 91%), disposable filtering facepieces (*n* = 18; 53%), elastomeric half- or full-facepiece respirators (*n* = 4; 12), shoe covers (*n* = 12; 35%), and hair bonnets (*n* = 12; 35%) (Figure 3B). In all cases, supplies of PPE were assigned regardless of the type of ENM used and the physical state of the material being processed. 

## 4. Discussion

This assessment provides some potentially useful information on the risk management practices currently used in some ENM workplaces worldwide. One limitation relates to the difficulty in gaining access to conduct on-site assessments and identifying the diverse range of workplaces where ENMs are manufactured or used, as only 45 companies voluntarily joined the survey, despite the 1740 invitations to participate. This low rate of response underlines the difficulties in achieving information from ENM companies that are generally not so available to disseminate data regarding their internal organization, as well as on health and safety practices. However, despite our lower rate of response, the number of respondent companies included in our survey (*n*. 34) results is comparable to those of other previously-published international surveys, such as the 45 US participating companies in Engeman et al. [12], the 35 enrolled in the US NIOSH industrywide survey of ENM manufacturers and users by Schubauer-Berigan et al. [15], as well as the 40 German and Swiss companies in Helland et al. [11]. Overall, this may certainly characterize a bias in interpreting the obtained results, as participating companies may represent those with a greater awareness concerning ENM risks and therefore be more responsible actors in developing/adopting preventive measures. This aspect may lead to an overestimation of the attention companies provide to the health and safety issues related to ENMs, without reflecting the real wide and varied ENM industry scenarios. This will require future assessments including a greater number of companies to obtain a more representative sample of the ENM-occupational scenario. From this perspective, company registry information regarding the quantity and type of ENMs produced and used may be employed to categorize target factories, but also informative campaigns aimed at increasing company awareness, concerning the need to provide information useful to support policies for health and safety management in the ENM field, may be extremely useful to increase survey participation.

On the other side, the low response rate may be due to the fact that ENMs are present only in few work processes/activities, and a small percentage of the total workforce in the companies is directly involved in such activities, therefore, limiting the interest the companies manifest on possible derived occupational risks and therefore to be enrolled in the survey. Additionally, the limited number of voluntary participating companies may not well reflect the diverse ENM industry. Moreover, workers were generally employed in small companies that may not be sufficiently representative of the real ENM occupational scenario, a further issue characterizing a possible bias in extrapolating information on the health and safety awareness in such an emerging industrial field. Despite the limitations, there are lessons to be learned from this assessment. Since the universe of workers exposed to ENMs is not well defined, any glimpse of practices might be beneficial to consider so that additional guidance materials may be developed. It is clear that the production and use of ENMs is growing and so will the number of ENM workers. Thus, it is necessary to reduce potential risks for workers expected to routinely handle ENMs and to define risk-management strategies adequate for exposure conditions experienced in each ENM life-cycle phase. 

This aspect may be of great relevance when defining health and safety programs suitable for both primary ENM producers and down-stream users who may experience completely different risks although derived from apparently similar hazards. In this regard, as a notable phase of the nano-product life, waste management practices require specific attention as they may both improve company nano-protective measures and also have external influences dealing with product end-of-life [12]. Forty-seven percent of the respondent companies reported procedures for the collection, storage, sorting, and disposal of waste products from ENM processing, a percentage greater than the 34% and 22% reported in private respondent companies that used and/or produced ENMs or in ENM research facilities [12,14], respectively.

Hazards of some ENMs have been identified in animal studies, but not all ENMs have been shown to be hazardous [18,19,20,21,22,23,24]. Consequently, it is important to consider each specific ENM in a workplace and obtain a suitable physico-chemical characterization. This activity was reported to be performed in less than half of the respondent companies. However, attention should be paid in interpreting characterization results. The survey, in fact, specifically referred to the activities of the enrolled companies and may not be able to unmask those facilities sending their material out for characterization. However, physico-chemical characterization should be encouraged to be routinely performed, at least in companies involved in development, production and direct application of ENMs, in order to better understand the toxicological behaviour of specific types of ENMs, as well as to correctly evaluate and manage risks for exposed workers. From a preventive perspective, this may be important to guide a safety-by-design approach to the synthesis and development of engineered ENMs [25,26]. Also, future investigation should clarify the possible impact of co-exposure to multiple ENMs and non-ENM hazardous conditions as reported by several companies in our assessment. 

Concerning risk management strategies, some items of the assessment were focused on understanding which informative sources may impact the “safety culture” of individual companies and influence risk assessment initiatives and precautionary measures. We observed that a great variety of resources, from industry and scientific conferences, to governmental materials, manufacturer information, and websites, have been indicated by more than half of the investigated enterprises. Moreover, such information sources had an impact on changing work processes and practices, including also the modification of work products, workplace health and safety procedures, as well as company policies, such as the updating of company policies for handling ENMs, as well as implementing training material [10]. However, more definitive references able to guide preventive initiatives specifically focused on ENMs seem necessary, as the majority of respondent companies reported that they adapted chemical risk-management guidance to nano-sized substances. This approach may be justified from the perspective of a precautionary risk-management strategy, however, its effectiveness needs to be carefully verified. 

In relation to the risk-management measures, the hierarchy of controls approach including the elimination/replacement of the most dangerous products; engineering exposure controls; administrative management measures, such as workforce training and information programs; as well as health surveillance plans and the adoption of PPE, were addressed [27]. Regarding the exposure assessment, 14 of the respondent companies (41% of the sample) indicated that they performed such measurements, but only 7 did on a regular schedule, while the others assessed environmental exposure only in the case of accidental events or when job processes were activated or somehow changed. These results may be dependent on the lack of definitive strategies to assess ENM exposure in terms of metrics, suitable techniques, and defined procedures [28]. 

The most implemented measure to control exposure to ENMs was the use of fume hoods, as also identified by Schubauer-Berigan et al. [15], Engeman et al. [12], and Diaz-Soler et al. [14], followed by local exhaust ventilation and HEPA filtration for exhausted air. These measures are commonly used as a standard control in workplaces involved in chemical activities; therefore, their effectiveness in reducing the levels of exposure to ENMs should be verified to support appropriate recommendations for future risk management [29]. Among other containment systems, the application of enclosed systems, i.e., glove boxes, was less-frequently adopted. In our assessment, their application occurred in 21% of the respondent companies, in line with the 21% and 30% reported by Diaz-Soler et al. [14] with respect to the type of organization considered in the analysis, i.e., University or Public Research agency, respectively, and lower than the 40% found by Schubauer-Berigan et al. [15]. As above detailed, in this latter survey, the percentage of companies that indicated using more-contained production, such as ventilated enclosures or glove boxes (40%), or completely enclosed (i.e., isolated) production processes (34%), resulted in being lower compared to the most commonly reported engineering controls among surveyed companies, such as chemical hoods (83%) and local exhaust ventilation (71%). Environmental spill cleanup procedures were adopted by 47% of the respondent companies, a higher percentage compared to that reported in Diaz-Soler et al. [14] (about 15% of the companies) and in Engeman et al. [12] (21%), which is maybe due to the variable job activities and processes performed in the different companies.

In our assessment, the vast majority of the respondent companies (85%) declared that their employees were provided with information on the safe use of ENMs. These data were in line with those reported by Engeman et al. [12], who showed that 90% of the investigated companies performed training courses for employees; while in the study by Diaz-Soler et al. [14], only 31% and 41% of workers received information and training about the risks posed by ENMs and control measures, respectively. However, caution should be raised in such comparison due to the possibility that different questionnaires employed in these studies may have focused on quite different aspects of the same item. In Engeman et al. [12], in fact, “training” generally referred to the programs through which companies communicated workplace safety and health practices and policies to their workforce [12]; on the other hand, in Diaz-Soler et al. [14], some differences have been shown between “training”, intended as information provided about the risks posed by ENMs, and “information” about protective measures to adopt while performing job tasks.

Only 12 companies reported health surveillance programs for their employees (35% of the respondent companies), while only 3 respondent companies (9% of the total) performed health surveillance specifically oriented at possible nano-risks for workers’ health. Overall, the health surveillance was generally poorly practiced, and its ENM-targeted application was even lower, as also reported in another investigation [14]. This finding may be due to the absence of specific guidelines as a consequence of the limited knowledge regarding the possible adverse effects of ENMs on workers. This gap should be thoroughly assessed in the future and supported by epidemiological and cohort studies focused on possible early alterations in exposed subjects [30]. 

With regard to PPE, gloves were the most common type, and respirators were reported in use in lower percentages as previously reported in other surveys [14,15]. In fact, companies manufacturing or using engineered carbonaceous materials in the US reported that gloves were the most widely-adopted form of PPE (89%), with slightly but non-significantly lower percentages (80%) reporting the use of respirators [15]. In Spanish research facilities performing tasks involving the use of ENMs at research level, nitrile gloves were reported as the most commonly employed PPE used. For airways protection, most of the respondents reported using the disposable filter mask FPP3, with respirators as a less frequently applied measure [14]. However, the choice of PPE did not generally consider the characteristics of the employed ENMs, the likelihood for environmental release, nor skin-absorption capabilities. Moreover, the effectiveness of PPE with respect to nanoscale chemical protection, the relationship between PPE adopted, and awareness about health and safety risks remain to be verified. 

Even if the limited number of companies make it challenging to point out differences in company characteristics and health and safety practices with respect to the company nationality and size, commercial fields of ENM application and type/quantities of ENMs employed, an attempt can be made to extrapolate few considerations comparing Italian companies (n. 8, 23% of the respondent sample) with respect to the data from the entire sample. In general, Italian companies are in line with the international scenario with regards to concerns of commercial sectors in which ENMs were primarily involved, such as professional technical or scientific services (50% of the Italian companies) and chemical fields (50% of the Italian companies), as well as the types of ENMs prevalently produced and/or used, e.g. metal-based ENMs. A general overlapping of data could also be determined for other explored fields, such as the developmental and instituted health and safety practices, exposure control measures, administrative controls, and personal protective equipment adopted. However, a greater percentage of the Italian companies (87%) indicated that they do not perform environmental monitoring evaluation in the workplace compared to the 59% of the total sample, suggesting the need to develop interventions to support the importance of such a procedure to assess and manage risks in ENM occupational settings.

## 5. Conclusions

This assessment characterized precautionary risk management currently followed by ENM companies. This was a small-scale assessment, but its value is that there is a limited amount of published literature on assessments of risk-management practice. Although there is the limitation related to the small number of respondent companies, the risk-management scenarios emerging from our survey appear in line with those described in other assessments available in the literature. From this perspective, all the published data may provide a comprehensive view of the challenging topic of risk assessment and management in the ENM occupational field. In general, industries involved in production and/or use of ENMs show a certain, although variable, awareness with regard to occupational health and safety protection, that seems more focused on general chemical risk management than on preventive actions specifically focused at nano-sized materials. Future assessments should include a greater number of companies to obtain a more representative sample of the ENM occupational scenario, and a direct access to the industrial settings with an “on site” collection of information should be pursued. From this perspective, company registry information regarding the quantity and type of ENMs produced and used may be employed to categorize target factories. Moreover, concerted action of involved stakeholders, from academia to industry, non-governmental organizations and regulators, workers’ representatives, and occupational health and safety professionals should be focused on developing standardized industrial frameworks for evaluating ENM risks with the aim of providing final guidance to nano-specific preventive actions. Finally, as more definite evidence becomes available on health and safety practices adopted in the ENM field, greater scientific efforts should be aimed not only at describing adopted risk-management policies, but also to define their effectiveness in protecting the health and safety of exposed workers.

## Figures and Tables

**Table 1:** Company features.

Company Nationality		Number of Respondent Companies
North America	Canada	1
Asia	India	2
	Iran	1
	Japan	1
	Thailand	1
Europe	Bulgaria	1
	Czech Republic	1
	Denmark	2
	Estonia	1
	Germany	2
	Greece	1
	Italy	8
	Norway	1
	Russia	1
	Spain	2
	Turkey	1
	U.K.	5
Not provided		2

**Table 2:** Summary of the changes introduced in health and safety policies according to NM risk awareness (n., number of respondent companies and relative percentages of the total survey sample).

Development/implementation of health and safety practices	Inform safety and health practices, *n*. 28 (82%) Inform company policies for handling ENMs, *n*: 24 (71%) Implement safety data sheets, *n*. 15 (44%) Modify work processes, *n*. 15 (44%) Implement training material, *n*. 14 (41%) Modify work products, *n*.9 (26%)
Production of informative material	No, company *n*: 5 (15%) Yes, company *n*: 29 (85%) In these latter cases, informative material included: Scientific publications, *n*. 18 (62%) Information provided to customers along with products or services, *n*. 16 (55%) Journal articles, *n*. 13 (45%) Instructions for how to use products or guidance for measuring nanoparticles, *n*. 10 (34%) Articles for trade magazines, *n*. 8 (28%) Information on standard procedures or practices in the company, *n*. 8 (28%) Comments on public policies public policies that are intended to be disseminated, *n*. 4 (14%) Comments or input on Government documents, *n*. 4 (14%) Comments or input to professional, scientific or trade associations, *n*. 4 (14%) Comments or input to industry or materials standards, *n*. 3 (10%)

**Table 3:** Summary of the instituted health and safety programs.

Instituted Health and Safety Programs in Investigated Companies	Number of Respondent Companies (%)
Determination of routes of exposure	16 (47%)
Identification of processes or job tasks where workers may be exposed	18 (53%)
Evaluation of new processes/procedures for hazards	17 (50%)
Review of purchase orders for possible hazardous materials	9 (26%)
Use of exposure controls (elimination, substitution, engineering, administrative, personal protective equipment)	19 (56%)
Assessment of effectiveness of exposure controls	7 (21%)
Assessment of need for personal protective equipment	14 (41%)
Maintenance of engineering controls (e.g., dust collection systems)	13 (38%)
Spill cleanup procedures	16 (47%)
Waste management/disposal procedures	16 (47%)
Medical screening and surveillance	3 (9%)
Exposure monitoring	11 (32%)
Systematic review and update of safe use procedures	14 (41%)
Method for reporting hazards, illnesses, and injuries	11 (32%)
Development of internal company exposure guidelines	11 (32%)
